# Fasciitis of the lower leg after COVID-19 vaccination

**DOI:** 10.1016/j.idcr.2022.e01475

**Published:** 2022-03-09

**Authors:** Satoshi Ide, Atsumasa Kurozumi, Akiko Takeshige, Akira Shimomura, Riri Watanabe, Takeshi Inagaki

**Affiliations:** a Department of General Internal Medicine, National Center for Global Health and Medicine, Tokyo, Japan; b Department of Cardiology, National Center for Global Health and Medicine, Tokyo, Japan

**Keywords:** COVID-19, Fasciitis, Vaccination

## Abstract

• The occurrence of fasciitis after COVID-19 vaccination is rare.
• The BNT162b2 COVID-19 vaccine can cause fasciitis distant to the injection site.
• The use of steroids is not essential for treatment of vaccine-associated fasciitis.

A 39-year-old man received a second dose of the BNT162b2 COVID-19 vaccine in the left deltoid muscle. Six days later, he presented with fever (38.5 °C), right leg pain, and difficulty walking. He reported no vigorous exercise or heavy manual labor prior to the symptom onset. Blood test revealed an elevated C-reactive protein (CRP) level (19.3 mg/dL) and white blood cell (WBC) count (15,100 cells/μL), with normal levels of creatinine kinase and myositis-related antibodies (antinuclear antibody, Jo-1, MDA5, Mi-2, TIF1g, RNA polymerase, and Scl-70). T2-weighted magnetic resonance imaging (MRI) showed high-intensity fascia in the right popliteal region and intermuscular fluid collection (Fig. 1). The patient was diagnosed with vaccine-related fasciitis and treated with loxoprofen. His leg pain resolved, CRP level and WBC count normalized, and MRI showed improvement. He was discharged 5 days later and showed no recurrence after 4 months.

**Fig. 1:**
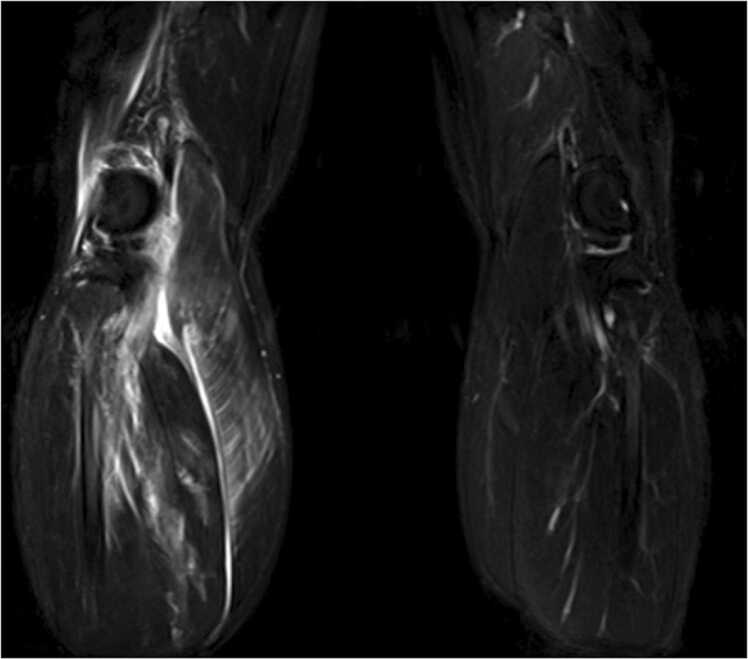
T2-weighted magnetic resonance imaging (MRI). High intensity image shows the fascia in the right popliteal region and intermuscular fluid collection.

Various vaccines have been developed against COVID-19 and are now available [1]. The COVID-19 vaccine is known to cause myocarditis, pericarditis, and myositis [2], [3]; however, the occurrence of fasciitis after COVID-19 vaccination is rare according to our literature search in PubMed as of February 16, 2022. There is a report of fasciitis in the lower extremities on the fifth day after the vaccination, akin to our case [4]. The late reaction is also known to occur with a median onset of day 8 after vaccination with systemic symptoms [5]. Also, fasciitis is caused by several etiologies, such as overload (e.g., plantar fasciitis), eosinophils, macrophages, monocytes, proliferation and degeneration (e.g., Dupuytren’s contracture), or bacterial infection (e.g., necrotizing fasciitis), however, none of above are suspected from patient history and course of the disease. Although no biopsy was performed, fasciitis is suspected to be a vaccine-related reaction for above reasons. Accumulation of similar reports are also necessary to establish delayed vaccination reaction. If the myalgia is severe, the MRI may be helpful to prove the presence of fasciitis.

Treatment of rhabdomyolysis and fasciitis after mRNA-1273 vaccine with high-dose steroids has been reported [4]. In comparison to our case, no renal dysfunction or elevated CK levels were seen. This case illustrates that the BNT162b2 COVID-19 vaccine can cause fasciitis distant to the injection site, and that the use of steroids is not essential. Clinicians should be made aware of fasciitis as a rare side effect after COVID-19 vaccination so that they may perform appropriate supportive therapy when needed.

## Ethical approval and Consent

Written informed consent was obtained from the patient for publication of this case report and accompanying images.

## Funding

This research did not receive any specific grant from funding agencies in the public, commercial, or not-for-profit sectors.

## CRediT authorship contribution statement

**Satoshi Ide:** Writing – original draft, Conceptualization. **Atsumasa Kurozumi:** Writing – review & editing. **Akiko Takeshige:** Writing – review & editing. **Akira Shimomura**: Writing – review & editing. **Riri Watanabe:** Writing – review & editing. **Takeshi Inagaki:** Writing – review & editing, Supervision. All authors contributed to the writing of the final manuscript.

## Conflicts of interest

The authors declare no conflict of interest.

## References

[1] Richman D.D. (2021). COVID-19 vaccines: implementation, limitations and opportunities. Glob Health Med.

[2] Oster M.E., Shay D.K., Su J.R., Gee J., Creech C.B., Broder K.R. (2022). Myocarditis cases reported after mRNA-based COVID-19 vaccination in the US from December 2020 to August 2021. JAMA.

[3] Nassar M., Chung H., Dhayaparan Y., Nyein A., Acevedo B.J., Chicos C. (2021). COVID-19 vaccine induced rhabdomyolysis: case report with literature review. Diabetes Metab Syndr.

[4] Faissner S., Richter D., Ceylan U., Schneider-Gold C., Gold R. (2021). COVID-19 mRNA vaccine induced rhabdomyolysis and fasciitis. J Neurol.

[5] Blumenthal K.G., Freeman E.E., Saff R.R., Robinson L.B., Wolfson A.R., Foreman R.K. (2021). Delayed large local reactions to mRNA-1273 vaccine against SARS-CoV-2. N Engl J Med.

